# The role of the thyroid in polycystic ovary syndrome

**DOI:** 10.3389/fendo.2023.1242050

**Published:** 2023-10-05

**Authors:** Huanhuan Fan, Qingling Ren, Zhejin Sheng, Ganxiu Deng, Limei Li

**Affiliations:** ^1^ Affiliated Hospital of Nanjing University of Chinese Medicine, Nanjing, China; ^2^ School of Life Science and Technology, Tongji University, Shanghai, China; ^3^ Department of Respiratory and Critical Care Medicine, Shanghai East Hospital, Tongji University School of Medicine, Tongji University, Shanghai, China; ^4^ Research Center for Translational Medicine, Key Laboratory of Arrhythmias of the Ministry of Education of China, Shanghai East Hospital, Tongji University School of Medicine, Shanghai, China

**Keywords:** pcos, thyroid, metabolic abnormalities, reproductive disorders, potential mechanism

## Abstract

Polycystic ovary syndrome (PCOS) is the most common endocrine and metabolic disease in women of childbearing age and can cause metabolic disorder, infertility, and increased anxiety and depression; as a result, it can seriously affect the physical and mental health of fertile women. PCOS is a highly clinically heterogeneous disease with unclear etiology and pathogenesis, which increases the difficulty of treatment. The thyroid gland has complex regulatory effects on metabolism, reproduction, and emotion, and produces hormones that act on almost all cells of the human body. The clinical manifestations of PCOS are similar to some thyroid diseases. Furthermore, some thyroid diseases, such as subclinical hypothyroidism (SCH), not only increase the incidence rate of PCOS, but also exacerbate its associated metabolic abnormalities and reproductive disorders. Interestingly, PCOS also increases the incidence of some thyroid diseases. However, the role of the thyroid in PCOS remains unclear. This review is intended to thoroughly explore the critical role of the thyroid in PCOS by summarizing the comorbidity of PCOS and thyroid diseases and their combined role in metabolic disorders, related metabolic diseases, and reproductive disorders; and by analyzing the potential mechanism through which the thyroid influences the development and progression of PCOS and its symptoms. We hope this review will provide a valuable reference for the role of the thyroid in PCOS.

## 1 Introduction

Polycystic ovary syndrome (PCOS) is the most common endocrine and metabolic disease in women of childbearing age, affecting 3–15% of women worldwide (1, 2). PCOS causes reproductive disorders, metabolic disorders, and psychological problems, all of which can seriously affect the physical and mental health of these women (3).

PCOS is a highly clinically heterogeneous disease; there are four specific phenotypes that vary greatly depending on life stage, genotype, race, and environmental factors. The four PCOS phenotypes are classified according to three factors: polycystic ovary morphology, ovulation dysfunction, and hyperandrogenemia (4). The etiology and pathogenesis of PCOS remain unclear, primarily due to the heterogeneity of these phenotypes, which increases the difficulty of treatment of this complex endocrine disease.

The clinical manifestations of PCOS are similar to some thyroid diseases. Increasing evidence shows that PCOS is related to the increased incidence rate of thyroid diseases, such as autoimmune thyroiditis (AIT), and subclinical hypothyroidism (SCH) (5–7). Palomba et al, have made a comprehensive narrative review on PCOS and thyroid disorder recently (8). This review provides us with a comprehensive understanding of the current research between thyroid and PCOS. However, we want to detail and discuss how alteration in thyroid function may influence PCOS to find the role of the thyroid in PCOS. This review intends to explore the important role of the thyroid in PCOS by analyzing the co-occurrence of PCOS and thyroid diseases; summarizing the incidences of metabolic disorders, related metabolic diseases, and reproductive disorders; exploring the possible mechanisms of interaction between PCOS and thyroid diseases; and ultimately providing a valuable reference for the role of the thyroid in PCOS.

## 2 Co-occurrence of PCOS and thyroid disease

The incidence rate of thyroid diseases is increased in patients with PCOS (9). In 2019, a study from Denmark reported that the risk of thyroid disease in PCOS patients is 2.5 times higher than that in patients without PCOS (10); therefore, we have thoroughly analyzed the comorbidity of several thyroid diseases with PCOS.

### 2.1 Hyperthyroidism

Graves’ disease (GD) is the primary clinical cause of hyperthyroidism with global incidences reported between 1.5 and 6.5 per 100,000 (11). It tends to increase in frequency with age. It is predominantly considered to be an organ-specific autoimmune disease (11), characterized by infiltration of the thyroid by T and B lymphocytes that react against thyroid antigens and the production of thyroid autoantibodies. The main thyroid autoantibodies are directed against thyroid-stimulating hormone receptors, which can activate the TSH receptor, leading to excessive production of thyroid hormones. Therefore, patients with this disease typically present with hyperthyroidism and diffuse enlargement of the thyroid gland (11).

There are few reports that indicate an association between PCOS and GD. Jung et al. was the first to describe a female patient with PCOS and GD in 2011; she presented with decreased menstruation, low body mass index (BMI: 16.4 kg/m^2^), mild hirsutism, and thyrotoxicosis (**Table 1**) (12). In 2012, six female patients with PCOS and GD were identified in a tertiary care center in northern India (13). They presented with goiter based on clinical and ultrasound examination; furthermore, all women were thin, with an average BMI of 22.73 kg/m^2^, and three of the six women had a waist circumference of < 80 cm. Additionally, UMA Sinha et al. reported two patients from India with both PCOS and GD, who showed elevated anti-thyroid peroxidase (TPO) antibody levels (14). The prevalence of PCOS combined with GD is unclear, because current information is limited to case reports, and extensive epidemiological data is currently lacking. Moreover, the incidence of PCOS combined with GD may differ with race or ethnicity, since the patients referenced in these case reports were all Asian women. The probability of having PCOS combined with dominant or subclinical hyperthyroidism in young female western populations is very low, which may be related to the lower prevalence of hyperthyroidism in the general western population (26).

**Table 1:** Comorbidity between PCOS and thyroid disease.

Participants		Country	Comorbidity between PCOS and thyroid disease	Ref
Study group	Control group			
18476 women with PCOS	54757 age-matched control	Danish	The event rate of thyroid disease was 6.0 per 1000 patient-years in PCOS Denmark versus 2.4 per 1000 patient-years in controls.	(10)
A 27-year-old female patient		South Korea	She was diagnosed as having PCOS and hyperthyroid Graves’ disease.	(12)
Six women		India	Six women presented with PCOS and Graves’ disease together.	(13)
80 PCOS patients	80 age-matched female subjects	India	Statistically significant higher prevalence of autoimmune thyroiditis (22.5% vs. 1.25% of control); high prevalence of goiter among PCOS patients (27.5% vs. 7.5% of control, P value > 0.001); higher percentage of PCOS patients (12.5%; controls 2.5%) had hypoechoic USG pattern.	(14)
5399 patients with GD	10,798 patients without GD	Taiwan, China	The adjusted hazard ratio for PCOS in patients with GD compared with patients without GD was 1.47 (95%CI = 1.09-1.98).	(15)
168 women diagnosed with PCOS		Brazil	A diagnosis of SCH was established in 11.3% of the women with PCOS.	(16)
200 females with PCOS	200 females without PCOS	Pakistan	SCH was found to be more prevalent in participant with PCOS compared to participants without PCOS (43.5% vs. 20.5%; p-value: <0.00001).	(17)
465 women with PCOS		Bangladesh	10.8% of them had SCH and 18.3% were positive for anti-TPO.	(18)
4821 participants		71.31% chinese patients out of the total	27.0% of them had SCH.	(19)
175 patients with PCOS	168 age-matched women without PCOS	Germany	Thyroid function and thyroid-specific antibody tests revealed elevated thyroperoxidase (TPO) or thyroglobulin (TG) antibodies in 14 of 168 controls (8.3%), and in 47 of 175 patients with PCOS (26.9%; P<0.001).	(20)
86 reproductive-age women diagnosed with PCOS	60 age-BMI matched control women	USA	HT was more common in PCOS patients compared to controls (22.1 and 5%; p = 0.004).	(21)
175 girls with euthyroid CLT (chronic lymphocytic thyroiditis)	46 age-matched non-CLT girls	India	Significantly higher prevalence of PCOS was noted in girls with euthyroid CLT when compared to their control counterparts (46.8 vs 4.3%, P=0.001).	(22)
97 women with PCOS	71 healthy female volunteers	Turkey	Twenty-nine patients with PCOS (29/97; 29.9%) had thyroid nodules, whereas only eleven control subjects had thyroid nodules (11/71; 15.5%) (p=.043).	(23)
178 patients with PCOS	92 age-BMI matched patients with no disease	Turkey	The number of nodules of 1 cm and above was found to be higher only in patients with PCOS compared with the control group.	(24)
6731 patients with AITD	26,924 controls	Taiwan, China	PCOS risk in patients with AITD was higher than that in the control group (adjusted hazard ratio = 1.39; 95% confidence interval = 1.07-1.71).	(25)

Recently, Chen et al. compared 5,399 patients with GD and 10,798 patients without GD in the Asian population (**Table 1**) (15). They found that the cumulative incidence rate of PCOS in patients with GD was significantly higher than that in patients without GD, with an adjusted risk coefficient for PCOS of 1.47 for GD patients compared with those without GD, indicating that women with GD may have an increased risk of developing PCOS. They also analyzed the comorbidities between GD and PCOS, including diabetes, obesity, hypertension, and heart failure. Only the incidence rate of hyperlipidemia was significantly increased in GD patients with PCOS, for the adjusted odds ratio of hyperlipidemia was 1.47 in patients with GD and was 2.18 in patients with GD and PCOS (15).

### 2.2 Subclinical hypothyroidism

The prevalence of SCH is higher in PCOS patients than that in the general population (16). Raj et al. conducted a study of Pakistani women 18–30 years old to determine the incidence of SCH in PCOS patients (**Table 1**) (17). By comparing 200 PCOS patients with 200 control patients without PCOS, they determined that SCH was more prevalent in PCOS patients (43.5%) than in participants without PCOS (20.5%). They also found that weight gain and BMI of the PCOS patients were significantly higher than those without PCOS (17).

Since hypothyroidism commonly occurs in PCOS patients, this correlation strongly suggests an increased risk of thyroid disease with PCOS; therefore, it is important to explain its clinical impact. SCH may cause mild metabolic abnormalities. For example, a clinical study of 4,065 PCOS patients 12–40 years of age revealed that more patients with SCH were shown to have obesity, central obesity, and goiter compared with the normal thyroid group (**Table 1**) (18). Furthermore, women with SCH are more likely to have abnormal fasting plasma glucose (FPG) levels and insulin resistance index (HOMA-IR) than women without SCH. Additionally, a study of 4,821 participants, comprised of 1,300 PCOS patients with SCH and 3,521 PCOS patients without SCH, found that the HOMA-IR, triglyceride, serum total cholesterol (TC), low density lipoprotein (LDL), fasting blood glucose (FBG), fasting C-peptide (FCP), and prolactin levels were higher, while high-density lipoprotein cholesterol (HDL), luteinizing hormone (LH), and testosterone levels were lower in the SCH patients (**Table 1**) (19). Collectively, these results indicate that the incidence of metabolic syndrome is higher in the SCH group, which indicates that SCH may aggravate lipid- and glucose-related metabolic disorders in PCOS patients.

### 2.3 Thyroiditis

Thyroiditis is a heterogeneous disease of the thyroid gland with various etiologies. Hashimoto’s thyroiditis (HT), also known as chronic lymphocytic thyroiditis, is a type of AIT and is a common form of thyroiditis in young women (27). HT may occur concurrently with clinical hypothyroidism (the most common), normal thyroid function, or hyperthyroidism.

Janssen et al. were the first to confirm that the prevalence of HT in PCOS patients was higher than that in non-PCOS patients through a systematic prospective study with 175 PCOS patients and 168 healthy controls (**Table 1**) (20). In 26.9% of PCOS patients and 8.3% of the control group, HT-specific anti-TPO or anti-thyroglobulin (TG) antibody levels were found to be elevated, revealing a threefold increase in the prevalence of HT in PCOS patients relative to controls. Furthermore, thyroid ultrasound examination results revealed that 42.3% of PCOS patients, but only 6.5% of the control group, exhibited a typical HT hypoechoic thyroid ultrasound pattern indicative of mild thyroid damage. Additionally, Arduc et al. conducted a study of women of childbearing age and compared 86 women with PCOS with 60 BMI-matched control women (**Table 1**) (21). Their results showed that the prevalence of HT in PCOS patients (22.1%) was higher than the control group (5%). Moreover, TSH was elevated in PCOS patients (26.7%) relative to controls (5%), indicating the presence of hypothyroidism in these PCOS patients with HT.

Since HT prevalence is known to be higher in women with PCOS, it is important to understand if the reverse is also true, namely, if the prevalence of PCOS is higher in women with HT than in women without HT. Ganie et al. conducted a prospective case-control study in India on adolescent females 13–18 years old comparing 1,075 HT patients with normal thyroid function and 46 age-matched patients without HT based on negative anti-TPO antibody tests (**Table 1**) (22). Their results showed that the prevalence of PCOS was significantly higher in HT patients (46.8%) than in non-HT patients (4.3%). Moreover, the BMI, waist circumference, and systolic blood pressure were all higher in HT patients than in controls.

### 2.4 Thyroid nodule

Thyroid nodules are discrete lesions within the thyroid gland, which can be detected by ultrasonography (28). The incidence rate of thyroid nodules in women is four times that in men, and its incidence rate increases with age and body mass index (29). They are typically benign. But about 5% of these lesions may ultimately be malignant (29). Therefore, the primary goal of thyroid nodule evaluation is to determine whether it is malignant. Generally, only nodules>1 cm should be evaluated as they are more likely to become clinically significant cancers. In rare cases, some nodules<1 cm may lead to future incidence rate and mortality (28). Each thyroid nodule has an independent risk of malignancy, and patients with multiple nodules may need biopsy on multiple nodules. Nodules with highly suspicious ultrasound features should be given priority for biopsy (29, 30).

Karaköse et al. analyzed 97 patients with PCOS and 71 healthy female volunteers as controls; they found that 29 PCOS patients (29.9%) had thyroid nodules, while only 11 control subjects (15.5%) had thyroid nodules (**Table 1**) (23). Furthermore, participants with thyroid nodules were older with higher fasting blood glucose, BMI, fasting insulin, and HOMA-IR values than participants without thyroid nodules, indicating an increased incidence of nodular goiter in PCOS patients. A retrospective study was conducted on 178 PCOS patients aged 18–45 years and 92 BMI-matched healthy control subjects (**Table 1**) (24). The PCOS group was higher than the control group in both the presence and number of nodules, including the number of nodules ≥ 1 cm. Further analysis of the PCOS patients showed that, relative to controls, phenotype A PCOS patients had the most prominent characteristics of thyroid dysfunction, such as increased thyroid autoimmunity, thyroid volume, and number of nodules > 1 cm, suggesting that thyroid dysfunction is more common in phenotype A of PCOS.

The incidence rate of thyroid disease is higher in PCOS patients than those without PCOS; conversely, the incidence rate of PCOS is higher in women with thyroid disease than in women with a normal thyroid. These analyses collectively indicate several clear characteristics of patients who have a combination of PCOS with thyroid disease: 1) Patients with combined diseases have more serious clinical manifestations than either disease individually; 2) Thyroid diseases associated with PCOS are primarily clinical hypothyroidism, including SCH, and autoimmune thyroid disease (AITD); and 3) Hyperthyroidism is rare with PCOS. Ho et al. found that the risk of PCOS in HT patients was 1.63 times higher than in those with normal thyroid function, while that in GD patients was 1.24 times higher (**Table 1**) (25), suggesting that hypothyroidism is more likely than hyperthyroidism to cause PCOS. In conclusion, there is a potential interplay between PCOS and thyroid disease—two common endocrine diseases in females—such that thyroid function affects the clinical and biochemical parameters of PCOS and, in turn, PCOS also affects thyroid function.

## 3 Thyroid disease exacrerbates metabolic disorders in PCOS patients

Women with PCOS are more likely to suffer from central obesity, T2DM, and dyslipidemia-associated metabolic syndrome than women without PCOS (31, 32).

### 3.1 Relationship between thyroid function and metabolic disorder in PCOS

The severity of metabolic abnormalities in PCOS patients is related to the degree of thyroid dysfunction. If thyroid hormone levels are too high, hyperthyroidism will appear, accompanied by symptoms such as overeating, hunger, and wasting. Conversely, if thyroid hormone levels are too low, hypothyroidism will occur (33).

Recent studies have reported that the incidence of hypothyroidism is higher in patients diagnosed with PCOS (11–14%) compared with control subjects (1–2%) (34, 35). Metabolic changes observed in both hypothyroidism and PCOS include insulin resistance, dyslipidemia, increased weight, and obesity (36). Compared with PCOS patients with normal thyroid function, women with PCOS and SCH combined have higher triglyceride levels, fasting insulin levels, and HOMA-IR (**Table 2**) (37). Furthermore, hypothyroidism often occurs in HT patients. Patients with combined PCOS and HT showed more severe metabolic symptoms than patients with PCOS or HT alone (5, 41). Females with combined HT and PCOS had higher BMI, fasting blood glucose, HOMA-IR, and cholesterol compared with the control group or HT group alone (21, 42). These findings suggest that the co-occurrence of PCOS and hypothyroidism is related to more significant metabolic and hormonal changes. The metabolic disorder in PCOS patients with HT-related subclinical and clinical hypothyroidism significantly improved with thyroid supplementation (43, 44), relative to those with normal thyroid function, which provides further evidence that the metabolic abnormalities seen in PCOS patients are related to thyroid dysfunction, which are improved after thyroid function returns to normal.

**Table 2:** Metabolic profile in PCOS with thyroid disease.

Participants		Metabolic profile	Ref
Study group	Control group		
114 PCOS patients with SCH	253 PCOS patients without SCH	Exacerbated the metabolic disorders (insulin resistance and dyslipidemia) in PCOS patients with SCH	(35)
20 patients with PCOS and SCH	39 patients with PCOS and normal thyroid function and 53 healthy women with normal thyroid function	Dyslipidemia with higher triglyceride levels and insulin resistance in group with PCOS and subclinical hypothyroidism	(37)
148 women with PCOS, without Type 2 diabetes mellitus (T2DM) and CVD present at baseline		At baseline, prevalent prediabetes was present in 18 (12%) of PCOS cases and it progressed to T2DM in 5 (3%) of the cases. Incident prediabetes during the follow-up was noted in 47 (32%) women or 4.7 per 1000 persons/year. cardiovascular risk in PCOS women with prediabetes was high.	(38)
100 females with PCOS	100 normal controls	Significantly higher HOMA-IR and frequency of subjects with dyslipidemia in SCH PCOS subjects	(39)
583 women with PCOS		Patients with elevated TSH levels had significantly increased fasting insulin and total cholesterol (TC)/high-density lipoprotein cholesterol (HDL) ratio.	(40)

In conclusion, thyroid function status directly affects metabolic disorders in PCOS patients. Generally, hypothyroidism will increase the metabolic burden of the body, leading to obesity and insulin resistance. Therefore, the thyroid function in PCOS patients must not be ignored, and efforts should be made to strengthen the detection of thyroid function.

### 3.2 Increased risk of T2DM complications with thyroid dysfunction in PCOS

The association between PCOS and T2DM has been fully confirmed. For example, a study monitored 148 PCOS patients for three years; initially, none of the patients had diabetes and 18 (12%) exhibited pre-diabetes symptoms (**Table 2**) (38). Over the course of the study, they found that 5 (3%) patients developed T2DM and 47 (32%) women developed pre-diabetes symptoms. This association is important to monitor, because the BMI of patients with pre-diabetes tends to increase, and the deterioration of glucose tolerance in PCOS patients may be accelerated.

Increasing studies have shown that hypothyroidism is associated with T2DM. Brenta et al. found that the prevalence of hypothyroidism in patients with T2DM was higher than in non-diabetic patients, and that diabetes complications in SCH patients were more common than those without SCH (45), which indicates hypothyroidism may be associated with T2DM. For every doubling of TSH, the incidence rate of T2DM increased 1.09 times. A large study of Danish PCOS patients (n = 19,199) showed that the prevalence of diabetes in Danish PCOS patients was higher than that in the control group (46). Moreover, compared with the control group, the incidence rate of thyroid disease was higher in the PCOS group, which indicates that thyroid disease may affect the incidence rate of diabetes complications in PCOS patients.

### 3.3 Dyslipidemia and risk of cardiovascular disease

Approximately 70% of PCOS patients exhibit a lipid metabolism disorder, including quantitative and qualitative changes in lipid mass spectra and lipoprotein parameters (16, 47). Compared with a PCOS group with normal thyroid function, the blood lipid profile of a PCOS group with SCH was significantly altered with a higher incidence of dyslipidemia (35, 39). SCH has been shown to adversely affect the lipid parameters of PCOS. In PCOS patients with SCH, lipid profiles are readily altered, resulting in increased LDL cholesterol (LDL-C) and triglyceride, and decreased HDL cholesterol (HDL-C) levels (**Table 2**) (40). Moreover, even in patients with PCOS and SCH with normal TSH levels, studies have still shown increased serum TC, LDL-C, non–HDL-C, and triglyceride levels with an associated decrease in HDL-C levels (48, 49), which indicates that SCH may increase the risk of cardiovascular disease in PCOS patients.

Women with PCOS are prone to dyslipidemia. The most common dyslipidemia features are hypertriglyceridemia, decreased HDL-cholesterol concentrations and the presence of small, dense LDL particles, which is characteristic of the atherogenic lipoprotein phenotype (50). Therefore, it is speculated that PCOS patients have a higher tendency to develop atherosclerosis.

In conclusion, patients with a combination of PCOS and thyroid disease have more severe metabolic abnormalities and may greatly increase risk of T2DM and cardiovascular disease than those with PCOS alone. The progression of metabolic disorders in PCOS can be mitigated to a certain extent by improving thyroid function.

## 4 Reproductive health disorders

Fertility disorders are one of the main characteristics of PCOS. The conception time of PCOS patients is significantly longer than that of the general population, and 40–70% of them are diagnosed with infertility, which is defined as the lack of conception after one year of routine unprotected sexual intercourse (51). Thyroid dysfunction also affects female reproduction; therefore, fertility problems may be more frequent and severe in patients with both PCOS and thyroid diseases, than in patients with thyroid diseases or PCOS alone (52).

### 4.1 Ovulation disorder, menstrual disorder, and fertility decline

Lack of ovulation is the most common type of PCOS-induced female infertility, and ovulation disorders are diagnosed in 75–85% of PCOS patients; only approximately 30% experience periodic ovulation (7, 53). Anovulatory infertility in women with PCOS is often associated with irregular menstruation (54). In adult women, both hypothyroidism and hyperthyroidism can cause menstrual disorder and fertility decline (7). Krassas et al. found that the levels of sex hormone binding globulin (SHBG), estradiol (E2), testosterone, androstenedione, LH, and follicle stimulating hormone (FSH) were higher in hyperthyroid patients than in those with normal thyroid function (55). Furthermore, the menstrual cycle of women with hyperthyroidism was irregular with abnormal ovulation relative to healthy women in the control group (56). Patients with hypothyroidism tend to have lower levels of SHBG, E2, testosterone, and androstenedione, and higher levels of prolactin than those with normal thyroid function (55, 56). In the pre-puberty stage, hypothyroidism may lead to delayed puberty (57); it may also lead to irregular menstruation, breakthrough bleeding, low endometrial thickness, ovulation dysfunction, and non-hyperplasia of endometrium due to anovulation (58).

All receptors in the thyroid hormone function signaling pathway, including thyrotropin releasing hormone receptor (TRHR), thyrotropin receptor (TSHR), and thyroid hormone receptors (TRs), have been detected in the monkey uterus and have been shown to be affected by steroid hormones (59), suggesting that the thyroid plays a regulatory role in female fertility. Some studies have shown that hypothyroidism may be related to the formation of ovarian cysts (60, 61).

### 4.2 Fertility risk

Compared with pregnant women with normal thyroid function, the spontaneous abortion rate of untreated SCH patients was higher than pregnant women with normal thyroid function (relative risk ratio (RR) = 1.90) (62). There was no significant difference between treated SCH patients and women with normal thyroid function (RR = 1.14), which indicates that hypothyroidism can increase the risk of spontaneous abortion.

Autoimmunity increases non-fertility risk. Antithyroid antibody is the most common autoimmune factor in infertile couples who have failed *in vitro* fertilization at least twice (63). The pooled results of multiple studies indicate that infertile women are more likely to develop AITD than the controls (64). Some studies have shown that AITD may lead to a 3–5-fold increase in the total abortion rate (65). Among the 438 women who received assisted reproductive technology, conception rates were similar between women with and without AITD, while women with AITD had significantly higher spontaneous abortion rates than women without AITD (53% and 23%, respectively) (66). Furthermore, SCH patients with thyroid autoimmunity have a significantly higher risk of miscarriage than other SCH patients (67, 68).

### 4.3 Maternal and infant health during pregnancy

PCOS and thyroid insufficiency also increase maternal and infant health risks during pregnancy, leading to pregnancy-related diseases. Compared with healthy controls, PCOS patients had an increased risk of maternal complications during pregnancy, including gestational diabetes, pregnancy induced hypertension syndrome (PIH), preeclampsia, premature birth, and increased need for a cesarean section during delivery (69, 70).

The risk of premature birth is closely related to PIH and obesity. Obesity also increases the mean time to conception (71). Elevated anti-TPO antibodies and the presence of both subclinical and dominant hypothyroidism can lead to infertility and adverse pregnancy outcomes, such as spontaneous abortion, preterm birth, small for gestational age (SGA), preeclampsia, stillbirth, cesarean section, and impaired fetal neurointellectual development (72). Therefore, women with combined PCOS and thyroid disease have a higher risk of infertility and pregnancy complications.

In conclusion, thyroid diseases, especially hypothyroidism and AITDs, exacerbate PCOS-related reproductive problems.

## 5 Analysis of potential thyroid-related etiologies of PCOS

Iodinated TG synthesized in follicular epithelial cells of the thyroid gland is decomposed by hydrolase to primarily form tetraiodothyronine, also known as thyroxine (T4), and a small amount of triiodothyronine (T3) (73–75). Maintaining homeostasis of circulating THs is the main function of the hypothalamic-pituitary-thyroid (HPT) axis (76); it is controlled by a highly specific system, in which iodothyronine deiodinases (DIOs) play a key role in tissue-specific hormone regulation (77, 78). Since the thyroid plays a key role in human metabolism, we specifically analyzed the possible role of the thyroid in PCOS development.

### 5.1 Elevated insulin resistance in the development of PCOS

**Figure 1:**
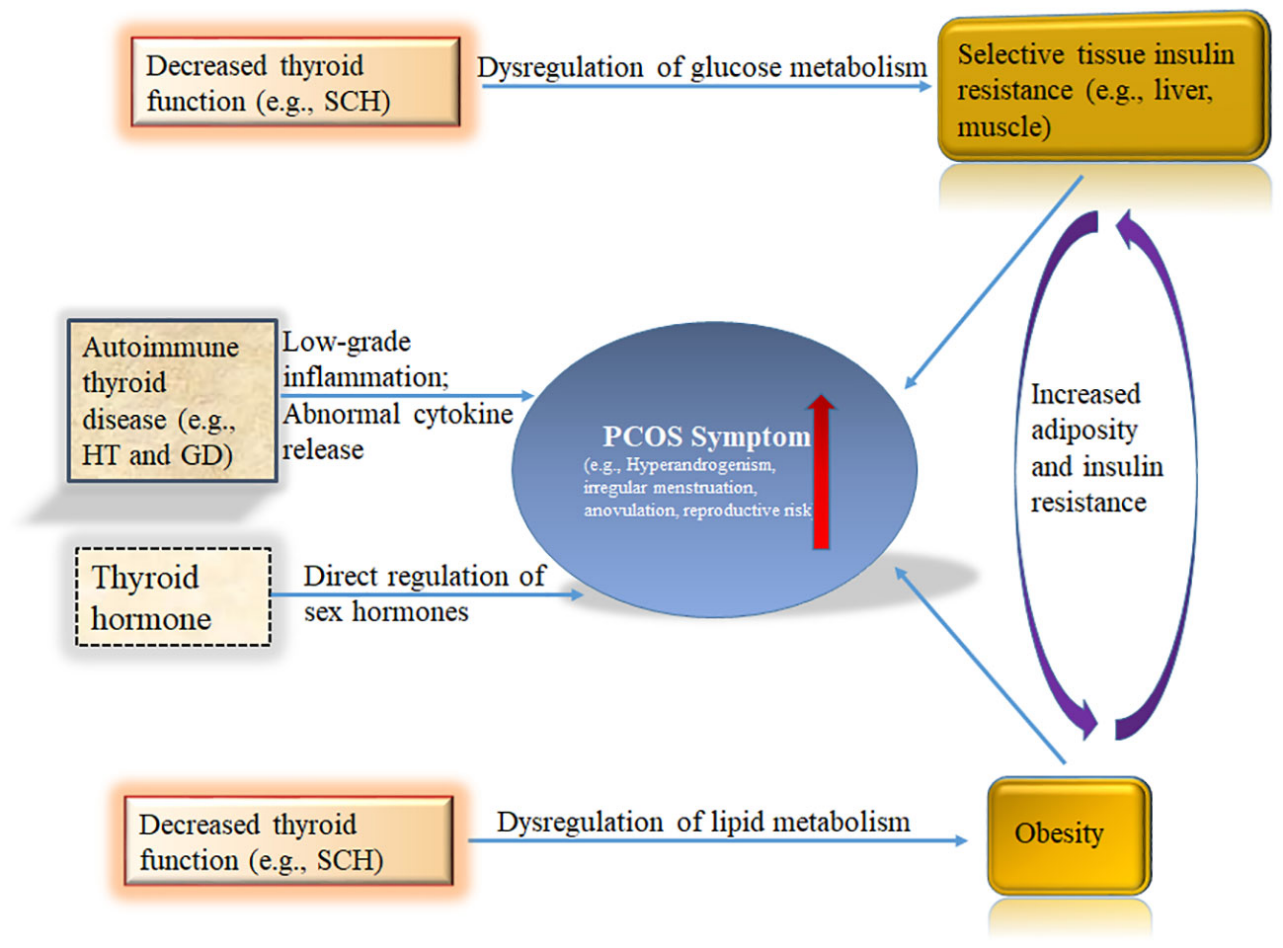
Schematic summary of the role of the thyroid in polycystic ovary syndrome (PCOS). Hyperinsulinism and obesity are necessary for full-blown pathogenesis of PCOS. Thyroid hormones participate in the regulation of blood glucose at the central and peripheral levels. Hypothyroidism has been shown to cause insulin resistance. THs regulate lipid metabolism. Decreased thyroid function readily leads to lipid metabolism disorders, including obesity. The immune state of the body also plays a key role in PCOS. HT and GD are common AITDs that have been repeatedly shown to increase PCOS risk in women. Sex hormones are regulated by Thyroid signal pathway. THs, Thyroid hormones; HT, Hashimoto’s thyroiditis; GD, Graves’ disease; AITD, autoimmune thyroid disease; SCH, subclinical hypothyroidism.

Insulin resistance and hyperinsulinemia play a key role in the development of PCOS, which in turn increases the risk of developing T2DM, irregular menstruation, and reproductive difficulties in PCOS patients (**Figure 1**) (79, 80). Regardless of BMI, the proportion of insulin resistance in PCOS patients is highly elevated compared with the general population. PCOS-related insulin resistance is characterized by decreased sensitivity and reactivity to insulin-mediated glucose utilization, primarily in skeletal muscle and adipose tissue (81). Significant internal factors are involved in the development of PCOS-related insulin resistance, although in some cases, it may only be acquired due to exogenous obesity. The root cause of PCOS is related to defects in intracellular insulin signaling downstream of the insulin receptor, which includes the hyperphosphorylation of serine residues on the insulin receptor substrate 1 (IRS-1) protein, which then binds to the insulin receptor and initiates insulin-specific intracellular responses (82–84). Insulin resistance has been observed not only in peripheral tissues of PCOS patients, such as adipocytes and skeletal muscle, but also in fibroblasts and ovarian granulosa and theca cells.

A study of PCOS patients compared BMI-matched PCOS patients with SCH (SCH-PCOS group) and PCOS patients with normal thyroid function, which revealed that the HOMA-IR of the SCH-PCOS group was significantly higher than that of the normal PCOS group (19). Additionally, the HOMA-IR in PCOS patients with normal thyroid function was significantly higher than that in the normal thyroid control group (85). Furthermore, it was found that the HOMA-IR of PCOS patients with hypothyroidism was the highest among all groups, and after receiving thyroid treatment, the HOMA-IR of PCOS patients with hypothyroidism decreased (86). These reports confirm the effect of hypothyroidism on carbohydrate metabolism parameters.

Thyroid hormones participate in the regulation of blood glucose at the central and peripheral levels (**Figure 1**). Centrally, T3 regulates glucose synthesis in the liver through the sympathetic nervous system (87). Peripherally, T3 enhances skeletal muscle glucose transporter 4 (GLUT-4) expression and downstream signaling cascade proteins responsible for glucose transport, which increases the insulin-dependent transmembrane glucose transport (88). Mueller et al. discovered a significant correlation between TSH—the most sensitive indicator of thyroid function—and insulin resistance, but not between insulin resistance and the patient’s age or BMI (89). Since hypothyroidism has been shown to cause insulin resistance, this relationship may explain how hypothyroidism could be an underlying factor for PCOS.

The compensatory hyperinsulinemia seen in insulin resistance is closely related to the anovulation seen in PCOS. For instance, the insulin resistance of ovulating PCOS patients is lower than that of PCOS patients with anovulation (90). All insulin level reduction treatments have been shown to improve ovarian dysfunction and ovulation (57, 91, 92). Significant insulin resistance is believed to be associated with hyperandrogenism and acanthosis nigricans (93). The compensatory hyperinsulinemia of insulin resistance may allow the ovaries to partially escape LH desensitization, resulting in increased responsiveness of ovarian steroids to LH (94, 95). Therefore, these studies raise the possibility that hyperinsulinemia may lead to ovarian androgen excess.

### 5.2 Lipid metabolism disorders and obesity in PCOS

THs regulate lipid metabolism. Decreased thyroid function readily leads to lipid metabolism disorders, including obesity (**Figure 1**) (33). Thyroid status is inversely related to blood lipid concentration. T3 regulates the activities of several key enzymes involved in lipoprotein transport, such as cholesterol ester transfer protein (CETP) and hepatic lipase (HL), thus regulating the distribution of HDL-C (96–98); furthermore, T3 may affect the synthesis and degradation of LDL-C. The LDL receptor gene promoter contains a T3 response element, which regulates the gene expression of LDL receptor C, thereby increasing the clearance rate of LDL-C (99). This thyroid-controlled regulation may explain why PCOS patients with hypothyroidism are prone to atherosclerotic cardiovascular disease.

PCOS patients are more prone to SCH; since obesity commonly occurs in PCOS patients with SCH, obesity may be a causative factor in the relationship between PCOS and SCH (**Figure 1**) (18). Since obesity leads to increased levels of leptin, this increased leptin could stimulate the hypothalamus and lead to increased TRH secretion (100). Additionally, DIO2 activity may be reduced through related proinflammatory conditions and insulin resistance, resulting in relatively low T3 and high TSH levels (101, 102). These possibilities could explain the high incidence of SCH in PCOS.

The influence of SCH on PCOS metabolic parameters is regulated by BMI. Mild thyroid hormone deficiency has no significant clinical impact on women with PCOS and low to normal BMI (103). However, overweight or obese PCOS patients will develop clinical symptoms when coupled with existing diseases, even if thyroid hormone deficiency is mild (103). Since PCOS symptoms may improve with weight loss, moderate weight loss (defined as a 5–10% reduction in initial weight) can improve many characteristics of PCOS, such as the insulin sensitivity index, and the hyperandrogenism (104, 105).

Recent evidence shows that excess adipose tissue is an important cause of excess androgen and estrogen production (106, 107). SHBG, also known as testosterone estradiol binding globulin, can regulate and control the concentration of active androgen, namely, free testosterone, thus causing hyperandrogenism symptoms (107). SHBG primarily binds to testosterone, but it also binds to dihydrotestosterone (DHT), androstenediol, estradiol, and estrone. Zhu et al. found a significant correlation between TSH and insulin sensitivity, insulin secretion, and SHBG, but only in the high BMI group (108).

In conclusion, PCOS is the most common obesity-related endocrine syndrome in women. The prevalence of obesity in PCOS case studies have been associated with race (109). At least one-third of normal weight PCOS patients exhibit abdominal obesity, while obese PCOS patients exhibit a more widespread distribution of adipose tissue (110, 111). Obesity produces testosterone through insulin resistance and circulating androstenedione, and inhibits the production of gonadotropin, which plays a role in PCOS (112).

### 5.3 Immune status in PCOS during some thyroid diseases

The immune state of the body also plays a key role in PCOS (**Figure 1**). In an Italian study, the prevalence of AITD in PCOS patients was significantly higher (26.03%) than in PCOS patients without other autoimmune diseases (9.72%) (113). Compared with Europeans and South Americans, the risk of AITD in women with PCOS is significantly higher among Asians (113). HT and GD are common AITDs that have been repeatedly shown to increase PCOS risk in women (**Figure 1**). HT, also known as chronic lymphoid thyroiditis and autoimmune thyroiditis, is an autoimmune disease characterized by increased serum gamma globulin and detection of thyroid autoantibodies in patients (27). Additionally, the thyroid tissue of HT patients exhibits lymphocyte infiltration, fibrosis, interstitial atrophy, and eosinophilic change of acinar cells. GD is also an autoimmune disease caused by sensitization of thyroid antigens by T lymphocytes, which stimulate B lymphocytes to synthesize antibodies against these antigens, thereby causing diffuse goiter with thyroid hyperfunction.

Sex hormones have immunomodulatory effects both *in vivo* and *in vitro*. In animal models, estrogen is associated with increased B cell activity and decreased T cell activity (114). The production of autoantibodies in female mice was higher than that in male mice. Estrogen can reduce the activity of suppressor T cells, increase the activity of B cells, increase the secretion of Th2 cytokine IL6, and guide the immune response to Th2 and the formation of antibodies (114). Compared with men, women have a higher CD4+/CD8+ ratio, CD4+ level, and antibody level (115). Androgen reduces most components of the immune system and enhances the activity of suppressor T cells, the Th1 response, and CD8+ activation (116). The above results indicate that estrogen in PCOS may exacerbate the production of autoantibodies caused by autoimmune thyroid diseases. In addition, progesterone can reduce the proliferation of macrophages, the synthesis of IL-6, and the production of peripheral antibodies (116). The fluctuation of progesterone concentration during the ovulation cycle and pregnancy is most likely associated with reversible changes of the immune system (117).

### 5.4 Sex hormones in PCOS patients may be misregulated by thyroid signal pathway

fT3 has been detected in follicular fluid (118, 119). The transcripts of TSH and its receptors not only exist in ovarian structures, such as oocytes, cumulus cells, granulosa cells, and the ovarian epithelium, but also in syncytiotrophoblast villi (120). Thyroid signal pathway receptors, including TRHR, TSHR, and TRs, have been identified in the monkey uterus, and estrogen and progesterone have been shown to affect their expression (59). TSH, TR α 1, and TR β 1 receptor expression have also been detected in human endometrium (121). The expression of TR α 1 and TR β were highest in the endometrium before ovulation. T3 regulates the synthesis of endometrial protein mRNAs, including leukemia inhibitory factor (LIF), which is important in the implantation process, and glucose transporter 1 (GLUT-1) (122). In addition, the placenta has T3 and T4 membrane transporters, DIO2 and DIO3 enzymes that regulate thyroid hormones (123). Oocytes and embryos cultured in a thyroid hormone-rich medium showed improved blastocyst formation, implantation ability, apoptosis rate, and vitality after cryopreservation (124). Therefore, the thyroid plays an important role in the reproductive system (**Figure 1**).

Significant hypothyroidism accompanied by elevated TRH led to hyperprolactinemia, interruption of pulsatile LH secretion, decreased SHBG synthesis, interruption of peripheral estrogen metabolism, and increased ovarian androgen production (125). In a study of female pigs, hypothyroidism led to increased gonadotropin receptor sensitivity in the ovaries, which ultimately promoted ovarian hypertrophy and the formation of multiple ovarian cysts (126). Moreover, hypothyroidism may lead to severe and irregular menstrual bleeding, spotting during the menstrual cycle, insufficient endometrial thickness, ovulation disorders, and eventually endometrial hyperplasia disorders (127, 128).

## 6 Conclusions and expectations

The clinical manifestations of PCOS are similar to some thyroid diseases. The thyroid diseases associated with PCOS are primarily SCH and AITDs; the co-occurrence of PCOS with hyperthyroidism is relatively rare. This association suggests that the thyroid may affect the clinical manifestations of PCOS by influencing multiple systems including metabolism and immunity. Insulin resistance is a widely recognized cause of PCOS, and thyroid function affects the degree of insulin resistance; specifically, hypothyroidism leads to more serious insulin resistance than hyperthyroidism. Obesity has seriously affected the health of modern people, and patients with hypothyroidism are prone to obesity. The patients with PCOS accompanied by hypothyroidism tend to have high BMI with a corresponding increase in metabolic disease burden. When the thyroid function of patients with SCH or clinical hypothyroidism is restored through thyroid treatment, their metabolic abnormalities improve (103). In addition, the thyroid also plays an important role in PCOS-related reproductive disorders. In conclusion, the role of the thyroid in PCOS is complex and involves multiple pathways. Thyroid function directly affects the clinical manifestations of PCOS, which increases the heterogeneity of the clinical PCOS phenotype. In fact, clinical reports have shown that some PCOS symptoms have been alleviated or even eliminated by restoring thyroid function (129). This evidence strongly suggests that the thyroid plays a crucial role in the pathogenesis, development, and progression of PCOS. Therefore, patients with PCOS require rigorous thyroid function detection, monitoring, and correction over time, which will mitigate or perhaps fully prevent the further deterioration of PCOS symptoms.

### 6.1 Limitation

Because the goal of this review is to find the role of the thyroid in PCOS, we focus on positive results between thyroid and PCOS. But there are quite a few negative data between thyroids and PCOS. Many factors are related to the above negative data, including the size of study populations, anthropometric parameters and follow-up period. For example, the diagnosis of PCOS has not yet been unified. Different studies have different diagnostic criteria for PCOS. Some studies require to exclude thyroid abnormalities, when PCOS is suspected. This will bring a wide variation in findings.

## Author contributions

All authors participated in the research and preparation of the manuscript. HF and QR contributed with the first draft of the manuscript, and actively participated in subsequent editing of the manuscript. ZS, GD and LL contributed with writing of subsequent versions and editing. LL and HF also did the final review and submission. All authors contributed to the article and approved the submitted version.
